# From GWAS signal to function: targeted CRISPR activation enables functional characterization of non-coding SNPs in chickens

**DOI:** 10.3389/fgeed.2025.1662152

**Published:** 2025-10-01

**Authors:** Jaewon Kim, Jeong Hoon Han, Minjun Kim, Grace Schmidt, Eunjin Cho, Jun Heon Lee, Tae Hyun Kim

**Affiliations:** ^1^ Department of Animal Science and Biotechnology, Chungnam National University, Daejeon, Republic of Korea; ^2^ Department of Animal Science, The Pennsylvania State University, University Park, PA, United States; ^3^ Department of Bio-AI Convergence, Chungnam National University, Daejeon, Republic of Korea; ^4^ The Huck Institutes of the Life Sciences, The Pennsylvania State University, University Park, PA, United States

**Keywords:** chickens, CRISPR activation, CRISPRa, cis-regulatory element, FAANG, GWAS, non-coding SNP

## Abstract

Genome-wide association studies (GWAS) have identified numerous single nucleotide polymorphisms (SNPs) associated with complex traits in poultry. However, most GWAS-identified variants reside in non-coding regions, making their functional relevance to their phenotypes unclear. Emerging evidence suggests that many of these markers overlap *cis*-regulatory elements, yet experimental validation of their biological function remains limited. Here, we investigated non-coding GWAS variants associated with nucleotide-related compounds in chicken breast muscle by targeting SNP-containing genomic regions using a CRISPR activation (CRISPRa) system in DF-1 cells and profiling transcriptomic responses via bulk RNA sequencing to assess the functional impact of activating these regions. Based on chicken muscle-specific epigenetic profiles and chromatin state annotations, we identified three significant GWAS variants on chromosome five associated with nucleotide metabolism. These variants are situated within *cis*-regulatory elements, specifically in intron three of *DUSP8*, intron one of *SLC25A22*, and upstream of *FBXO3*. To understand their functional impact, we employed an *in vitro* CRISPRa system with targeted guide RNAs to activate each non-coding SNP region in DF-1 cells. This activation resulted in significant changes at the transcriptomic level. Subsequent functional enrichment analysis of the differentially expressed genes consistently highlighted muscle-related pathways across all SNPs, including MAPK signaling, cytoskeletal remodeling, and ECM–receptor interactions, which are potentially involved in regulating nucleotide metabolism and deposition in muscle. Furthermore, transcript-level analysis of RNA-seq reads revealed that the non-coding SNP region within the intron three of *DUSP8* may function as an alternative promoter, resulting in significantly higher expression of a shorter transcript that could generate a non-canonical protein isoform. Our study demonstrates that activating genomic regions harboring specific non-coding GWAS SNPs can modulate gene expression, suggesting that these SNPs may contribute to gene regulatory functions. Importantly, this work underscores the powerful utility of CRISPRa as a functional genomics tool for linking GWAS signals to their biological roles in chickens by targeting SNP-containing regions and uncovering consequential molecular phenotypes.

## 1 Introduction

Genome-wide association studies (GWAS) have been widely employed to identify genetic variants associated with complex traits by analyzing millions of single nucleotide polymorphisms (SNPs) (Tan et al., 2023). In poultry, GWAS has been actively applied to uncover genomic loci associated with economically important traits such as growth, meat quality, egg production, and disease resistance (Smith et al., 2020; Zhang et al., 2021; Kim et al., 2023; Wang et al., 2024). These studies have provided critical insights into the genetic architecture of key traits and have laid the foundation for effective genomic selection strategies in the poultry industry. SNPs located within protein-coding regions can directly alter amino acid sequences or affect codon usage, making it relatively straightforward to predict their functional consequences (Claussnitzer et al., 2020). However, over 90% of SNPs identified by GWAS are located in non-coding regions, presenting major challenges in understanding their biological significance (Tak and Farnham, 2015). Many of these non-coding variants have often been dismissed as mere markers in linkage disequilibrium (LD) with causal variants or as artifacts of population structure (Li and Keating, 2014). However, recent studies have shown that a substantial proportion of disease-associated non-coding SNPs reside within *cis*-regulatory elements (CREs), such as promoters and enhancers, which modulate gene expression in a tissue-specific manner and influence phenotypic traits (Alsheikh et al., 2022; Pandey et al., 2024). Therefore, elucidating the regulatory roles of non-coding SNPs identified through GWAS is essential for uncovering the biological black box underlying complex quantitative traits and advancing precision breeding strategies in livestock.

Meat flavor plays a critical role in shaping consumer preference and has become an increasingly important target in poultry breeding programs (Takahashi, 2018; Huang et al., 2022). In chicken, flavor primarily develops during cooking through thermal reactions involving various precursor compounds, including nucleotides, free amino acids, peptides, and other nitrogen-containing substances (Jayasena et al., 2013). Among these, nucleotide-related compounds (e.g., inosine 5′-monophosphate (IMP), inosine, and hypoxanthine) are widely recognized as key contributors to the umami taste of meat (Dashdorj et al., 2015). These compounds are synthesized in muscle via *de novo* purine biosynthesis and the salvage pathway. After slaughter, adenosine triphosphate (ATP) is sequentially degraded to IMP, inosine, and hypoxanthine, resulting in a transient accumulation of IMP. This *postmortem* process is influenced by factors such as enzyme activity, pH decline, and muscle temperature, which collectively determine the residual levels of flavor-related nucleotides in meat (Huang et al., 2022). GWAS have identified several genes associated with nucleotide compound content as potential genetic markers (Uemoto et al., 2017; Kim et al., 2023). To further elucidate the biological basis of meat flavor, recent research has moved beyond GWAS by employing multi-omics strategies, such as transcriptomics and metabolomics, to uncover the genes and pathways involved in the metabolism of nucleotide-related compounds (Gai et al., 2023; Huang et al., 2023; Yu et al., 2023). However, epigenomic approaches have not yet been employed to investigate the regulatory mechanisms controlling these genes in muscle tissue. Given that the synthesis and metabolism of nucleotide-related compounds are controlled by gene expression, signal transduction, and complex regulatory networks, identifying upstream epigenetic regulators represents a critical next step.

Bridging the gap between statistical association and biological function requires the identification of epigenomic features, a goal that has motivated large-scale efforts to annotate regulatory elements in livestock genomes. In this context, the Functional Annotation of Animal Genomes (FAANG) consortium has made significant contributions by generating high-resolution maps of regulatory elements across diverse tissues in livestock species, including chickens (Giuffra et al., 2019). In chickens, the FAANG project has cataloged tissue-specific regulatory landscapes by profiling transcriptomes, chromatin accessibility, and histone modifications across a range of key tissues and organs (Kern et al., 2021; Pan et al., 2023). Integration of epigenetic datasets has revealed that SNPs identified through GWAS and selection signature analyses are predominantly enriched within putative regulatory elements, suggesting that many of these variants may exert their effects through transcriptional regulation (Pan et al., 2023; Shen et al., 2024). Despite these advances, functional studies that leverage epigenomic annotations to validate the roles of non-coding SNPs remain limited. Moreover, while these chromatin annotations are generated through advanced statistical models leveraging complex multi-omics data, experimental validation in chickens is essential to confirm their reliability and reproducibility (Mendieta et al., 2021; Foroozandeh Shahraki et al., 2024). This underscores the importance of empirical approaches to verify the regulatory functions of non-coding regions.

High-throughput methods such as massively parallel reporter assay (MPRA) and self-transcribing active regulatory region sequencing (STARR-seq) have recently been proposed to investigate the potential regulatory functions of non-coding regions identified through GWAS; however, these approaches primarily focus on measuring regulatory activity and do not fully capture downstream biological effects that influence phenotypes (Bruner and Grant, 2024; Chin et al., 2024). CRISPR-based approaches complement these techniques by enabling direct assessment of gene regulatory effects. In particular, the CRISPR activation and interference (CRISPRa/i), which utilize a nuclease-dead Cas9 (dCas9) fused to transcriptional activator or repressor domains, allow locus-specific modulation of gene expression through targeted guide RNAs (gRNAs) (Gilbert et al., 2014). Building on this framework, our previous study demonstrated that the CRISPRa system in the DF-1 chicken fibroblast cell line is a robust and effective platform for validating tissue-specific enhancers and promoters in the chicken genome (Chapman et al., 2023; Han et al., 2023).

**FIGURE 1:**
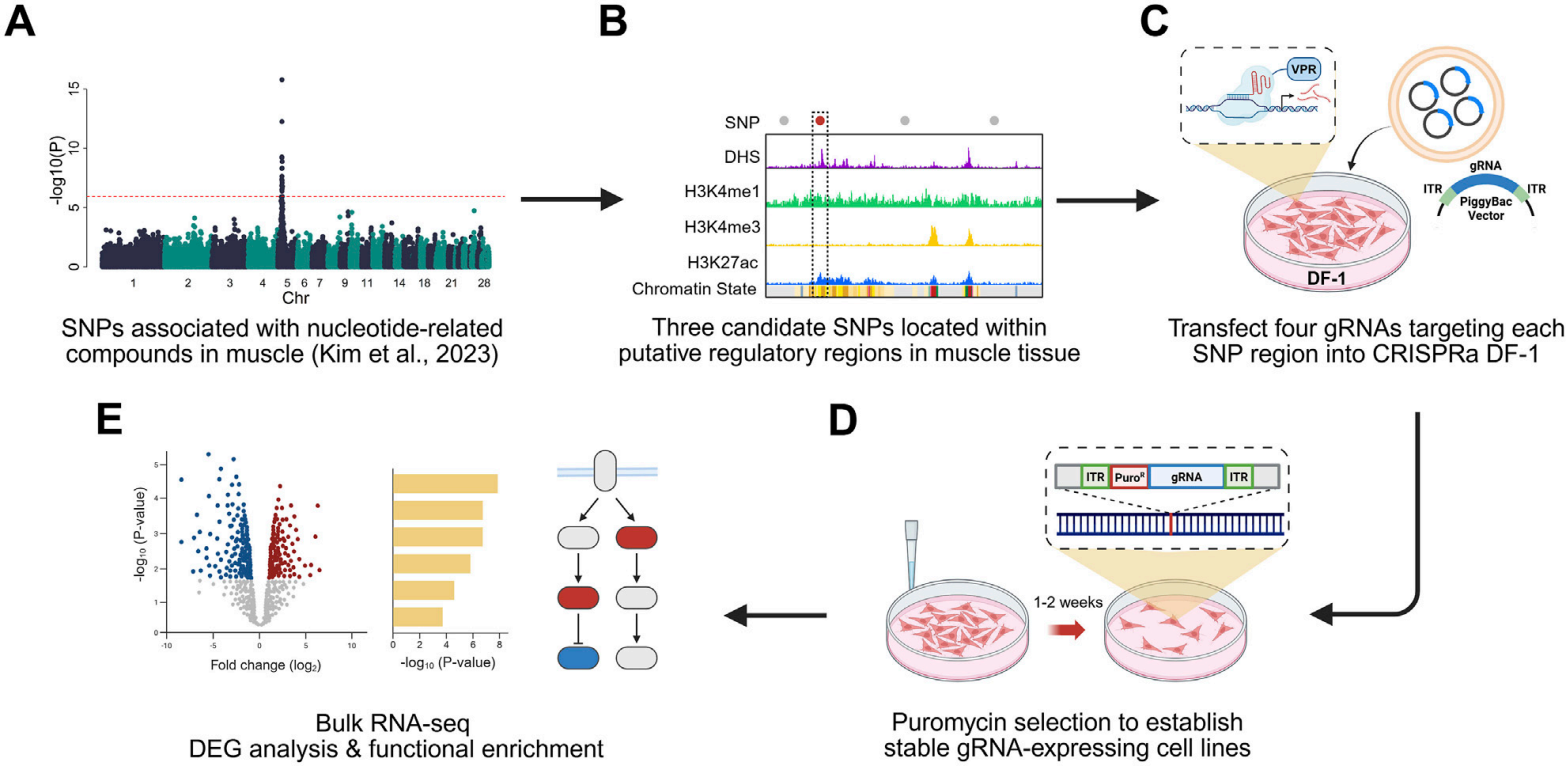
Experiment scheme of CRISPRa-based functional validation of non-coding SNP regions. **(A)** Non-coding SNPs associated with nucleotide-related compounds in chicken breast muscle (Kim et al., 2023) were identified through genome-wide association studies (GWAS). **(B)** Non-coding SNPs located within putative regulatory elements were selected based on regulatory chromatin marks, including DNase hypersensitivity site (DHS), H3K4me1, H3K4me3, and H3K27ac. **(C)** Three candidate SNPs overlapping putative regulatory elements were selected and targeted using a dCas9-VPR–based CRISPR activation system in chicken fibroblast (DF-1) cells. For each SNP regions, four gRNAs were designed and transfected. **(D)** Stable gRNA-expressing cell lines were generated through puromycin selection. **(E)** Differential expression and functional enrichment analyses using bulk RNA sequencing data confirmed the regulatory potential of the non-coding regions. Created with BioRender.com.

In this study, we aimed to functionally validate GWAS-identified non-coding SNPs associated with nucleotide-related compounds in chicken breast muscle (Figure 1). We employed a CRISPRa (dCas9-VPR) system to activate candidate regulatory regions and used RNA-seq to assess transcriptomic changes. This approach enabled us to identify putative regulatory elements and investigate the potential roles of these GWAS-linked loci in modulating muscle-related gene expression and purine metabolism.

## 2 Materials and methods

### 2.1 Identification of SNPs in putative regulatory elements associated with nucleotide-related compounds in chicken breast meat

We used 47 SNPs that were significantly associated with the content of two nucleotide-related compounds, IMP and inosine, in chicken breast muscle, based on our previous GWAS using 60K SNP chips (Bonferroni-adjusted P-value <1.15 × 10^−6^) (Kim et al., 2023). To annotate putative regulatory elements, DNase I hypersensitive site sequencing (DNase-seq), chromatin immunoprecipitation sequencing (ChIP-seq) data for H3K4me3, H3K27ac, and H3K4me1, and RNA sequencing (RNA-seq) data from chicken muscle tissue were obtained from the Functional Annotation of Animal Genomes (FAANG) repository (Kern et al., 2021). Using the *intersect* function in BEDTools (2.26.0) (Quinlan, 2014), we identified SNPs overlapping with DNase-seq and H3K27ac peaks in chicken muscle tissue annotated by Kern et al. (2021) (Kern et al., 2021). Among the 47 candidate SNPs, three (rs316338889, rs313523098, and rs317345807) were prioritized based on their overlap with putative regulatory regions and their statistical significance in the GWAS. These SNPs were subsequently designated as GW1, GW2, and GW3, respectively (Supplementary Table S1). Tissue-specific chromatin state annotations and Ensembl chicken regulatory features were further utilized to assess the epigenomic context of each SNP region (Pan et al., 2023; Dyer et al., 2024).

### 2.2 Establishment of DF-1 CRISPRa cell line

Chicken DF-1 fibroblast cells (CRL-12203; American Type Culture Collection (ATCC), Manassas, VA, United States) were cultured in Dulbecco’s Modified Eagle Medium (DMEM; Hyclone, Logan, UT, United States) supplemented with 10% fetal bovine serum (FBS; Thermo Fisher Scientific) and 1× antibiotic-antimycotic solution (Thermo Fisher Scientific, Waltham, MA, United States). Cultures were maintained at 37 °C in a 5% CO_2_, humidified incubator (60%–70% relative humidity). We generated a DF-1 cell line with CRISPR activation capability by applying a genome engineering approach utilizing CRISPR/Cas9 and homology-directed repair (HDR). CRISPR/Cas9 vectors were designed to target the 3′downstream region of the chicken *GAPDH* gene (GAPDH#1) and were constructed using the PX459 backbone (pSpCas9 2A-Puro; Addgene #62988, a gift from Feng Zhang). To ensure consistent transgene expression and controlled copy number, CRISPRa elements were inserted into the *GAPDH* locus via HDR-mediated targeted integration. A custom-constructed SP-dCas9-VPR expression vector containing left and right homology arms (0.4–0.6 kb each) flanking the transgene cassette was used as the CRISPRa donor. Using Lipofectamine 2000 (Thermo Fisher Scientific) according to the manufacturer’s instructions, DF-1 cells were co-transfected with 1.5 µg of the SP-dCas9-VPR donor plasmid and 1.5 µg of the GAPDH#1 gRNA construct (F: 5′-CAC​CGA​GCA​TCT​CTA​GTA​ACA​AAG​G-3′, and R: 5′-AAA​CCC​TTT​GTT​ACT​AGA​GAT​GCT​C-3′) (Chapman et al., 2023). Puromycin (1 μg/mL) was added 24 h post-transfection, followed by Geneticin (G418; 300 μg/mL) at 72 h. Cells were maintained under G418 selection for approximately 4 weeks to establish a stable CRISPRa DF-1 cell line.

### 2.3 gRNA design and vector cloning

gRNAs were designed for each putative regulatory region using the CHOPCHOP algorithm (https://chopchop.cbu.uib.no/) (Montague et al., 2014). For each locus, four gRNAs were selected to span approximately 500 bp around the SNP, with at least one gRNA directly overlapping the variant site. A mock control gRNA, which does not match any sequence in the chicken genome, was used as a negative control. A gRNA-expressing vector, used in a previous study (Chapman et al., 2023), was utilized. The plasmid included a gRNA scaffold regulated by the human U6 promoter and a puromycin resistance cassette driven by the human TK promoter. The vector was digested with the restriction enzyme BbsI (New England Biolabs, Ipswich, MA, United States), and gRNAs targeting each region, as well as the mock control, were inserted by ligation (Cong et al., 2013). Successful insertion of each gRNA was confirmed by Sanger sequencing. The gRNA sequences are listed in Supplementary Table S1. Potential off-target sites for each gRNA were predicted using CRISPOR algorithm (Concordet and Haeussler, 2018), with only sites containing up to three mismatches considered in the analysis.

### 2.4 Transfection of gRNA vector

CRISPRa DF-1 cells were plated in 12-well plates 1 day before transfection, allowing the cultures to reach 70% confluency at the time of transfection. For each target region (GW1, GW2, and GW3), a set of four gRNA vectors was co-transfected using Lipofectamine 2000 (Thermo Fisher Scientific) according to the manufacturer’s instructions. A total of 3 µg of plasmid DNA was prepared for each transfection by combining four gRNA vectors (600 ng each) targeting each region with 600 ng of a piggyBac transposase expression plasmid (PB200; System Biosciences, Palo Alto, CA, United States). This plasmid mixture was diluted in 100 µL of Opti-MEM (Thermo Fisher Scientific) and then mixed with 3 µL of Lipofectamine 2000 in an additional 100 µL of Opti-MEM. Transfections were performed in three biological replicates per target region. After 24 h, the culture medium was replaced with growth medium containing puromycin (1 μg/mL), and cells were maintained under selection for at least 9 days to ensure stable genomic integration of the gRNAs.

### 2.5 RNA isolation and bulk RNA-seq analysis

Total RNA was extracted from 12 samples, consisting of three biological replicates for each SNP-targeted region (GW1, GW2, and GW3) and the mock control, using the Direct-Zol RNA Miniprep Kit (Zymo Research) according to the manufacturer’s protocol. Bulk RNA-seq libraries were prepared using the NEBNext Ultra II RNA Library Prep Kit (New England Biolabs) and sequenced on the Illumina NovaSeq X Plus platform, generating over 20 million 150-bp paired-end reads per sample. Raw sequencing reads were assessed for quality using FastQC (0.12.1) (https://www.bioinformatics.babraham.ac.uk/projects/fastqc/) and trimmed using TrimGalore (0.6.10) (Krueger, 2012). Trimmed reads were aligned to the chicken reference genome GRCg7b (GCF_016699485.2) using STAR (2.7.11b) (Dobin et al., 2012), and gene-level read counts were obtained with HTSeq (2.0.5) (Anders et al., 2014). Aligned bam files were visualized using Integrative Genomics Viewer (IGV) (2.17.4) (Thorvaldsdóttir et al., 2012) to inspect gene expression patterns at selected loci. DESeq2 (1.40.2) (Love et al., 2014) was used to identify differentially expressed genes (DEGs) by comparing each CRISPRa-targeted group (GW1, GW2, and GW3) individually to the mock control. Genes with fewer than 10 total read counts across all samples were excluded prior to analysis, and the apeglm shrinkage method was applied to stabilize fold change estimates (Zhu et al., 2018). Genes with a false discovery rate (FDR) less than 5% were considered differentially expressed. Gene Ontology (GO) enrichment and Kyoto Encyclopedia of Genes and Genomes (KEGG) pathway analysis of DEGs were performed using DAVID (Huang et al., 2007). In addition, transcript-level quantification was carried out using Salmon (1.10.3) (Patro et al., 2017) in quasi-mapping mode. The decoy sequences and transcriptome index were constructed using the GRCg7b reference genome (GCF_016699485.2). Transcript-level TPM values were imported into DESeq2 via the tximport package (1.36.0) (Soneson et al., 2015) in R and used for normalization and expression comparison. DUSP8 amino acid sequences and domain information were obtained from the NCBI database, including the Conserved Domain Database (CDD) (Marchler-Bauer et al., 2014). Structural modeling of DUSP8 isoform proteins was performed using AlphaFold3 (Abramson et al., 2024).

## 3 Results

### 3.1 Selection of GWAS SNPs associated with nucleotide-related compounds through epigenomic data integration

**FIGURE 2:**
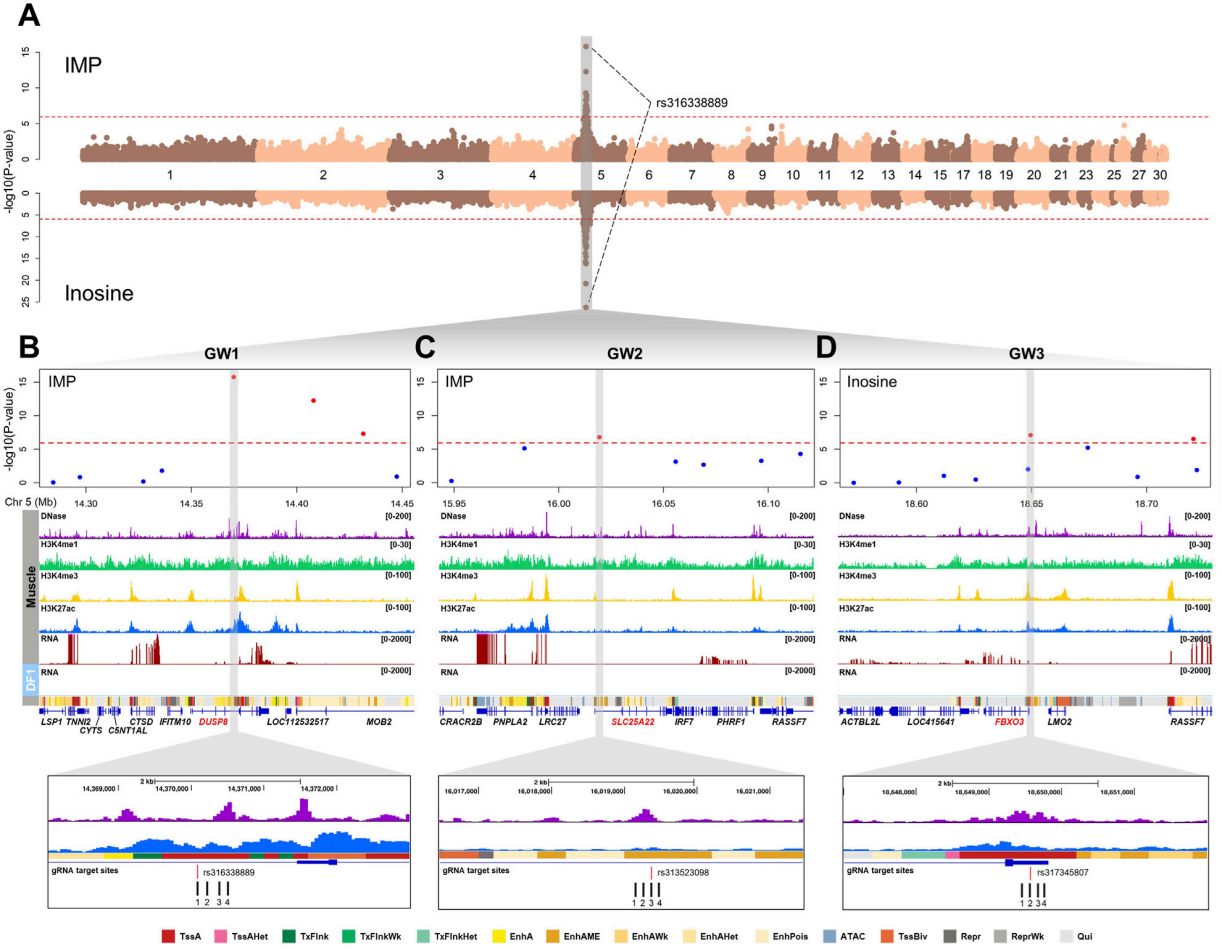
Genomic and epigenomic landscape of candidate non-coding SNPs associated with nucleotide-related compounds in chicken muscle. **(A)** Manhattan plots showing GWAS results for IMP (top) and inosine (bottom) content in chicken breast muscle. The red dashed lines represent the Bonferroni-corrected 5% genome-wide significance threshold (P < 1.15 × 10^−6^). All genome-wide significant SNPs, including rs316338889 associated with both traits, were located on chromosome 5. **(B–D)** Regional plots of GW1 (rs316338889) **(B)**, GW2 (rs313523098) **(C)**, and GW3 (rs317345807) **(D)** loci, showing chromatin (DNase-seq, H3K4me1, H3K4me3, and H3K27ac) and transcriptomic signals in chicken muscle tissue and DF-1, and predicted muscle-specific chromatin states. Gray-highlighted regions correspond to putative regulatory elements overlapping with non-coding SNPs, with gRNA target sites indicated below. TssA, strongly active promoter; TssAHet, flanking active TSS without ATAC; TxFlnk, transcribed at gene; TxFlnkWK, weak transcribed at gene; TxFlnkHet, transcribed region without ATAC; EnhA, strong active enhancer; EnhAMe, medium enhancer with ATAC; EnhAWK, weak active enhancer; EnhAHet, active enhancer without ATAC; EnhPois, poised enhancer; ATAC, ATAC island; TssBiv, poised TSS; Repr, repressed polycomb; PeprWk, weak repressed polycomb; Qui, quiescent.

Our previous study identified 47 significant SNPs associated with the contents of nucleotide-related compounds (IMP and inosine) in breast meat (Bonferroni-adjusted P-value <1.15 × 10^−6^), with rs316338889 showing the strongest association across all three compounds on chromosome 5 (Figure 2A) (Kim et al., 2023). Annotation of these SNP locations revealed that all these SNPs were positioned in non-coding regions, including introns, untranslated regions (UTRs), and intergenic sites. To further narrow down potential regulatory SNPs that may influence gene expression, we integrated chicken muscle epigenomic data, including DNase I hypersensitivity sites and ChIP-seq profiles for H3K4me1, H3K4me3, and H3K27ac. Through this integrative approach, we selected three SNPs (rs316338889, rs313523098, and rs317345807) that overlapped with putative regulatory elements, and designated them as GW1, GW2, and GW3, respectively (Figures 2B–D). GW1 and GW2 were associated with both IMP and inosine contents, while GW3 was associated only with inosine (Supplementary Table S1). According to the FAANG regulatory feature track in the Ensembl chicken genome annotation (GRCg7b, release 113), GW1 was positioned within an epigenetically modified accessible region and was located adjacent to a predicted enhancer element. Similarly, GW2 was located within an epigenetically modified accessible region, whereas GW3 was found to reside within a promoter region (Supplementary Figure S1). Based on the predicted chromatin states of chicken muscle tissue annotated using Hidden Markov Model by Pan et al. (2023), the region containing GW1 was marked as an active promoter (TssA) based on high emission probabilities for DNase-seq, H3K27ac, and H3K4me3 (Figure 2B). In contrast, the chromatin region containing GW2 was annotated as a medium enhancer (EnhAME) due to its high DNase-seq emission probability and moderate levels of H3K27ac and H3K4me1 (Figure 2C). GW3 was annotated as TssA, consistent with the FAANG regulatory feature annotation (Figure 2D). To assess the regulatory function of predicted *cis*-regulatory elements at the three loci (GW1, GW2, and GW3), we designed four gRNAs to target each element. For each locus, at least one gRNA was designed to directly overlap the associated SNP (Figures 2B–D).

### 3.2 Targeted activation of GW1 and its impact on the transcriptome

**FIGURE 3:**
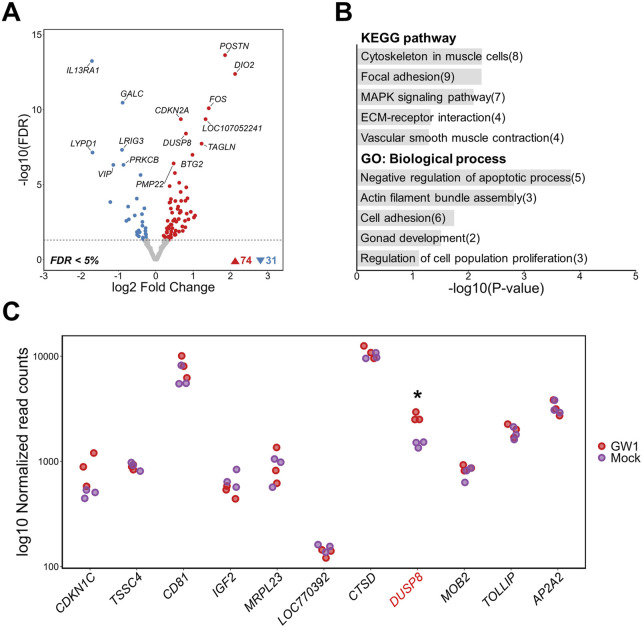
Transcriptomic changes following activation of a putative regulatory region containing GW1 (rs316338889) in DF-1 cell lines. **(A)** Volcano plot showing differentially expressed genes (DEGs) between GW1-activated and mock control DF-1 cells (FDR <0.05). **(B)** Top 5 KEGG pathway and GO biological process enrichment analyses of DEGs. Numbers in parentheses indicate the number of DEGs associated with each term. **(C)** Gene expression changes within a 2 Mb window surrounding the GW1. The gene harboring GW1 (*DUSP8*) is shown in red font. Red and purple dots represent normalized gene expression levels of CRISPRa-targeted (GW1) samples and mock controls, respectively. Significant differential expression indicates as * (FDR <0.05).

GW1, the most statistically significant SNP identified through GWAS, is located within the intron three of the *DUSP8* gene and is strongly associated with inosine (Bonferroni-adjusted P-value = 5.83 × 10^−27^) and IMP (Bonferroni-adjusted P-value = 1.62 × 10^−16^) contents (Supplementary Table S1). The chromatin region harboring GW1 exhibited strong enrichment of active regulatory markers, including DNase-seq accessibility, H3K4me3, and H3K27ac signals (Figure 2B). To investigate the regulatory potential of this putative element and its impact on gene expression, we activated the region surrounding GW1 using a dCas9-VPR system, followed by bulk RNA-seq analysis. As a result of activating the GW1 region, a total of 105 DEGs were identified, including 74 upregulated and 31 downregulated genes (FDR <0.05). Notably, *DUSP8* was among the upregulated genes (Figure 3A; Supplementary Figure S2). To better understand the transcriptomic changes and the associated biological pathways of the DEGs, we conducted functional enrichment analysis. KEGG pathway analysis revealed that the DEGs were significantly enriched in muscle-related pathways, including “Cytoskeleton in muscle cells”, “Vascular smooth muscle contraction”, “Focal adhesion”, “MAPK signaling pathway”, and “ECM-receptor interaction.” GO analysis of biological processes further revealed enrichment in terms related to cytoskeletal structure and cell survival, such as “Actin filament bundle assembly” and “Negative regulation of apoptotic process” (Figure 3B). Collectively, these findings suggest that the non-coding GW1 region may function as a regulatory element influencing muscle-related pathways. In many GWAS, researchers commonly prioritize genes located near identified significant SNPs as candidate genes for further study (Cai et al., 2018; Kong et al., 2025). This strategy relies on LD between causal variants and nearby SNPs, as well as the assumption that causal variants may exert *cis*-regulatory effects on neighboring genes (Cano-Gamez and Trynka, 2020). Following this rationale, we examined gene expression within a 2 Mb window surrounding the GW1 region to detect potential *cis*-regulatory effects. Of the genes in this region, only *DUSP8* showed significant differential expression, while the others showed no significant changes (Figure 3C). Off-target prediction confirmed that the gRNAs used for GW1 activation did not produce any significant off-target effects, further supporting the regulatory role of the GW1 region in modulating *DUSP8* expression (Supplementary Table S9).

### 3.3 Targeted activation of GW2 and its impact on the transcriptome

**FIGURE 4:**
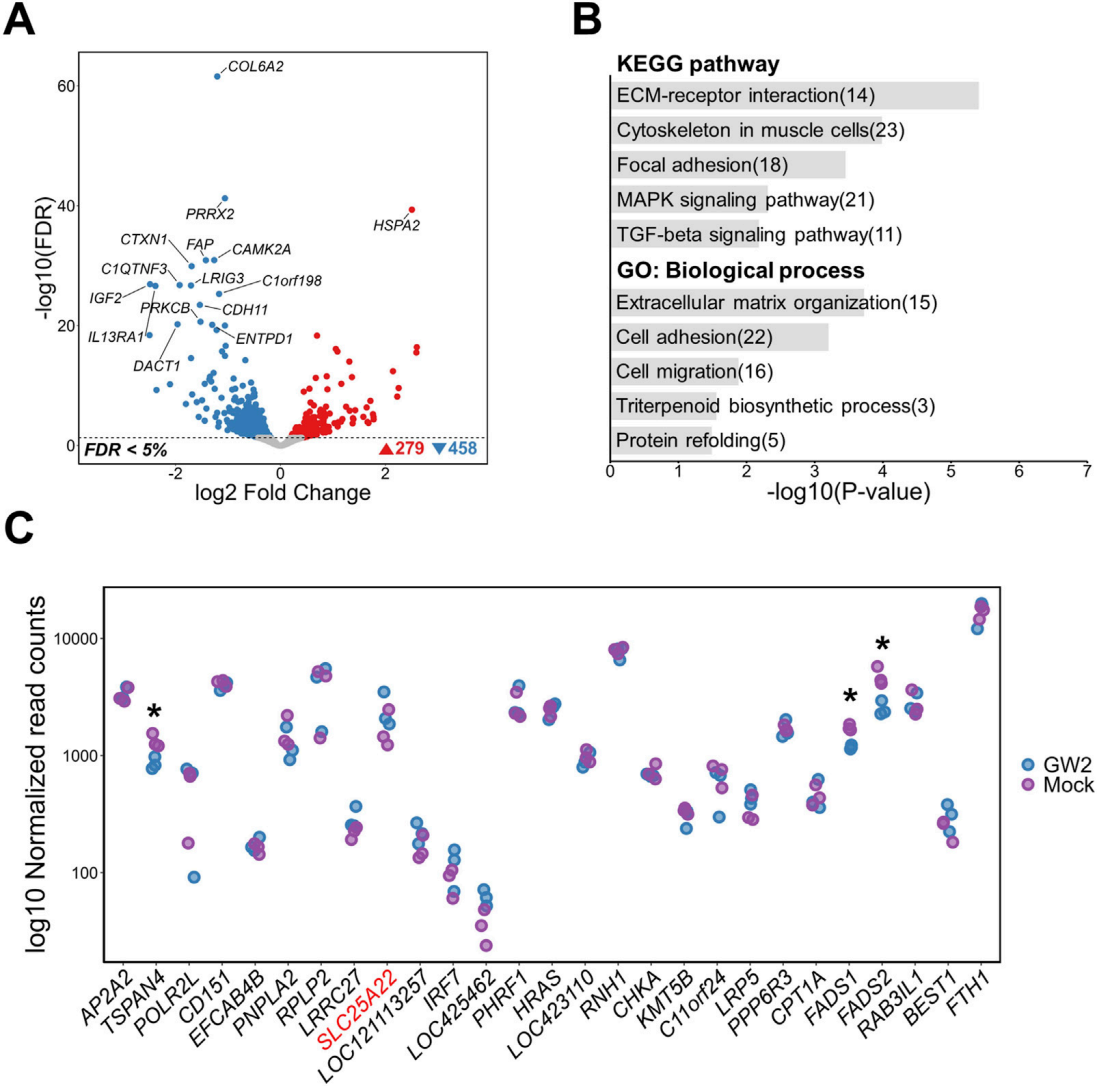
Transcriptomic changes following activation of a putative regulatory region containing GW2 (rs313523098) in DF-1 cell lines. **(A)** Volcano plot showing differentially expressed genes (DEGs) between GW2- activated and mock control DF-1 cells (FDR <0.05). **(B)** Top 5 KEGG pathway and GO biological process enrichment analyses of DEGs. Numbers in parentheses indicate the number of DEGs associated with each term. **(C)** Gene expression changes within a 2Mb window surrounding the GW2. The gene harboring GW2 (*SLC25A22*) is shown in red font. Blue and purple dots represent normalized gene expression levels of CRISPRa-targeted (GW2) samples and mock controls, respectively. Significant differential expression indicates as * (FDR <0.05).

The chromatin state surrounding GW2 was predicted to be a moderate enhancer (Figure 2C). Although this region exhibits relatively lower H3K27ac and H3K4me1 signals compared to strong enhancers, it remains an accessible enhancer capable of binding transcription factors and potentially contributing to gene regulation. Transcriptome-wide analysis identified 737 DEGs, with 279 upregulated and 458 downregulated transcripts (FDR <0.05) as a result of GW2 activation (Figure 4A). Functional enrichment analysis of these DEGs revealed transcriptional signatures related to cellular architecture, adhesion, and extracellular matrix remodeling. Significantly enriched KEGG pathways included “ECM-receptor interaction” and “Cytoskeleton in muscle cells.” GO biological processes highlighted structural and organizational programs such as “Extracellular matrix organization,” “Cell adhesion,” and “Cell migration” (Figure 4B). Activation of GW2 did not lead to significant upregulation of nearby genes within a 2 Mb window, including *SLC25A22*, which contains GW2 within one of its introns. However, *TSPAN4*, *FADS1*, and *FADS2* were significantly downregulated in GW2-activated samples compared to mock controls (FDR <0.05) (Figure 4C). For GW2, although seven potential off-target sites with two mismatches were identified, no significant changes in the expression of genes located near these off-target sites were observed. This suggests that these off-target sites did not contribute to the transcriptomic changes observed in response to GW2 activation (Supplementary Tables S9, 10).

### 3.4 Targeted activation of GW3 and its impact on the transcriptome

**FIGURE 5:**
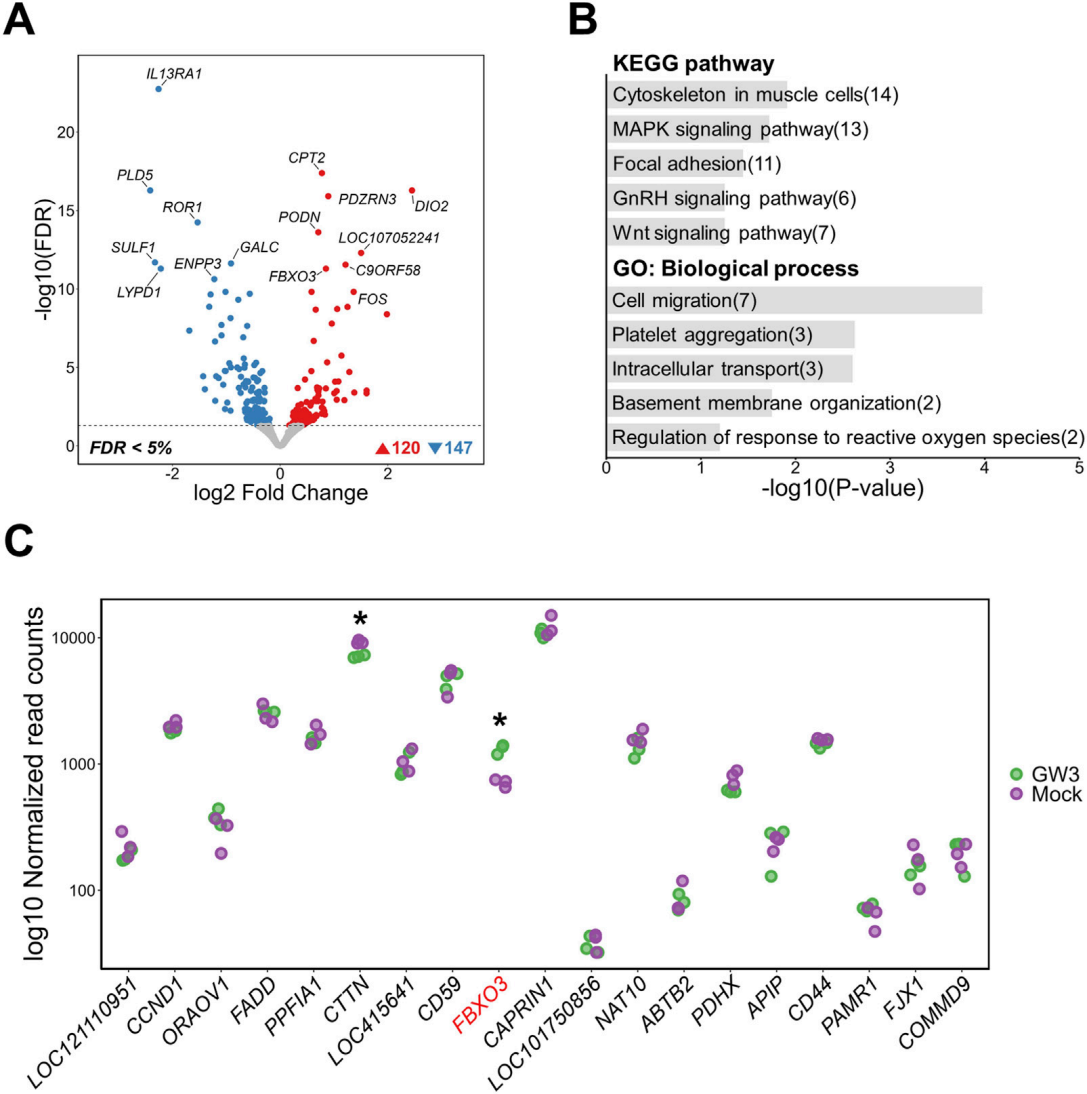
Transcriptomic changes following activation of a putative regulatory region containing GW3 (rs317345807) in DF-1 cell lines. **(A)** Volcano plot showing differentially expressed genes (DEGs) between GW3-activated and mock control DF-1 cells (FDR <0.05). **(B)** Top 5 KEGG pathway and GO biological process enrichment analyses of DEGs. Numbers in parentheses indicate the number of DEGs associated with each term. **(C)** Gene expression changes within a 2Mb window surrounding the GW3. The gene harboring GW3 (*FBXO3*) is shown in red font. Red and purple dots represent normalized gene expression levels of CRISPRa-targeted (GW3) samples and mock controls, respectively. Significant differential expression indicates as * (FDR <0.05).

GW3 is located within a region predicted to function as an active promoter in chicken muscle tissue, near the transcription start site (TSS) of the *FBXO3* gene (Figure 2D). Upon GW3 activation, genome-wide transcriptomic analysis identified 267 DEGs, including 120 upregulated and 147 downregulated genes compared to mock activation (FDR <0.05) (Figure 5A). Notably, *FBXO3* was among the most significantly altered genes, supporting the regulatory relevance of GW3 within the promoter region (Supplementary Figure S2). Functional enrichment analysis showed that DEGs were associated with a broad range of cellular processes, including structural pathways such as “Cytoskeleton in muscle cells” and “Focal adhesion,” as well as signaling-related pathways including “MAPK signaling pathway,” “GnRH signaling,” and “Wnt signaling.” GO biological process terms were associated with cellular structure and activity, including “Cell migration,” “Intracellular transport,” “Basement membrane organization,” and “Regulation of response to reactive oxygen species” (Figure 5B). Within a 2 Mb window around GW3, *FBXO3* was significantly upregulated in GW3-activated samples, while *CTTN* showed a reduction in expression (Figure 5C). Similarly, off-target prediction for the gRNAs used to activate the GW3 region did not show any significant off-target effects, supporting the specificity of the CRISPRa-mediated perturbation at this region (Supplementary Table S9).

### 3.5 The GW1 region may function as a promoter for a non-canonical *DUSP8* transcript

**FIGURE 6:**
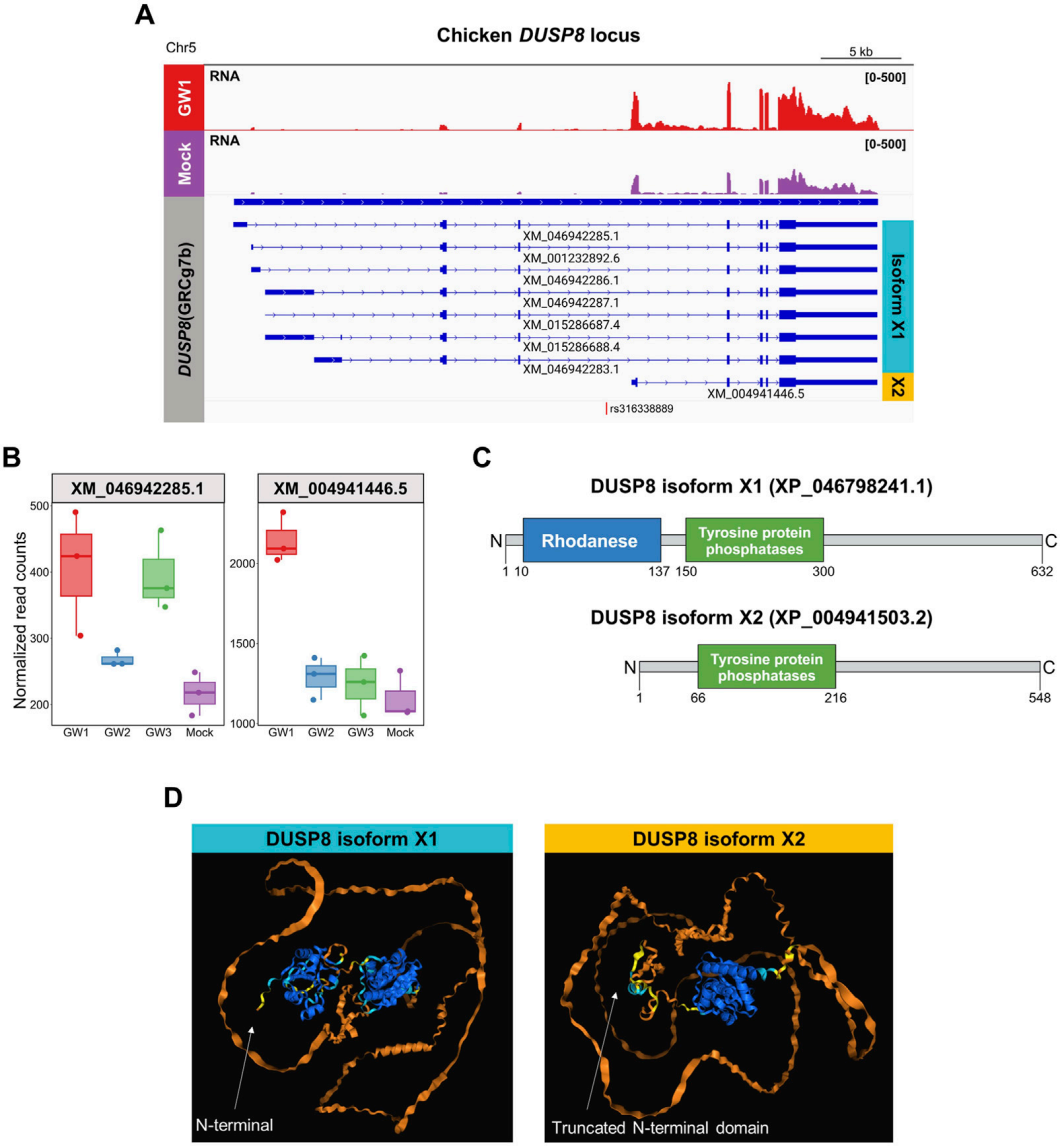
Transcript abundance and predicted protein isoform structures of chicken *DUSP8*. **(A)** Genome browser track of RNA-seq read coverage at the *DUSP8* locus in GW1-activated and mock control DF-1 cells. Annotated transcript isoforms are predicted to encode two protein variants: isoform X1 (cyan) and isoform X2 (yellow). Location of GW1 is indicated at the bottom. **(B)** Transcript-level expression comparison between the long (XM_046942285.1) and short (XM_004941446.5) isoforms of *DUSP8* across GW1 (rs316338889), GW2 (rs313523098), GW3 (rs317345807) and mock controls. **(C)** Domain structures of DUSP8 isoforms. Full-length DUSP8 (isoform X1) contains a Rhodanese domain (blue) and Tyrosine-protein phosphatase domain (green), whereas the atypical DUSP8 (isoform X2) lacks the Rhodanese domain. **(D)** Predicted 3D protein structures of DUSP8 isoforms X1 and X2. The structure is colored by predicted local distance difference test (pLDDT) confidence scores: blue (very high, pLDDT >90), cyan (confident, 90 > pLDDT >70), yellow (low, 70 > pLDDT >50), and orange (very low, pLDDT <50).

In the chicken reference genome (GRCg7b), *DUSP8* is annotated with eight transcript isoforms, including seven long isoforms (e.g., XM_046942285.1, XM_015268687.4) and one short isoform (XM_004941446.5). The GW1 region is located within the intron three of the long isoforms but lies near the TSS of the short isoform. We hypothesized that the GW1 region may serve as an alternative promoter for *DUSP8*, and that its activation may affect expression of specific mRNA isoforms. To test this, we first examined RNA-seq read coverage across the *DUSP8* locus and found that a notable increase in read counts was observed over the exons specific to the short isoform in GW1-activated samples (Figure 6A). Transcript-level quantification confirmed that short isoform exhibited the most pronounced increase in expression following GW1 activation, whereas the long isoforms, including XM_004941446.5, showed marginal or no specific changes. GW3 activation increased the expression of the long DUSP8 isoform without affecting the short isoform, suggesting that the upregulation of the long isoform is mediated through distinct downstream biological pathways rather than direct cis-regulatory control within the DUSP8 locus. In contrast, GW2 activation did not significantly alter the expression of either isoform, indicating that the short isoform induced by GW1 activation is specific to GW1 activation and is not influenced by other regulatory elements or pathways (Figure 6B; Supplementary Table S8). To investigate the potential function of the shorter transcript, we translated its mRNA sequence and found that it maintained a conserved reading frame relative to the canonical *DUSP8*. Protein sequence analysis revealed that all long mRNA isoforms encode the same DUSP8 protein (XP_046798241.1), whereas the short isoform (XP_004941503.2), translated exclusively from the shorter transcript, lacks the rhodanese domain (Figure 6C). Structural modeling using AlphaFold3 corroborated this difference, predicting both protein domains in isoform X1 with high confidence, while the N-terminus of isoform X2, translated from the short transcript, appeared truncated but retained an intact C-terminal domain (Figure 6D).

## 4 Discussion

This study aimed to provide a proof of concept demonstrating that the CRISPRa toolkit can be used to functionally investigate GWAS-identified non-coding SNPs in chickens. Here, we employed a dCas9-VPR-based system to activate non-coding GWAS SNP regions associated with muscle nucleotide composition in chicken breast muscle and assessed changes in gene expression and biological pathways to elucidate the potential functions of these variants.

The three non-coding SNP regions (GW1, GW2, and GW3) located in putative regulatory elements were selected for CRISPRa activation based on epigenetic datasets (DNase-seq and ChIP–seq) from chicken muscle tissue. For each selected region, we compared two sources of regulatory annotations: (1) the Ensembl FAANG regulatory database (Dyer et al., 2024), and (2) chromatin state predictions from a previous study (Pan et al., 2023). GW3 was consistently annotated as a promoter in both the Ensembl FAANG database and the chromatin state predictions from Pan et al. (Pan et al., 2023), supporting its potential role as a proximal regulatory element. In contrast, GW1 and GW2 were broadly identified as open chromatin regions, yet exhibited inconsistent regulatory classifications between the two datasets. These discrepancies underscore the complexity of regulatory element classification, and suggest that reliance on predictive annotations alone may be insufficient. Therefore, our findings highlight the necessity of experimental functional studies and support the regulatory potential of the selected SNP regions. Furthermore, given that the GWAS dataset used in this study was derived from a low-density SNP chip, the identified significant SNPs may not be the causal variants themselves, but could instead be in strong LD with nearby functional variants (Kindt et al., 2013). Based on this assumption, we hypothesized that causal variants may reside within the putative regulatory regions flanking these SNPs. Alterations in the DNA sequences of non-coding regulatory elements, caused by such variants, can influence transcriptional activity by modifying the binding affinity of transcription factors that interact with these elements (Chin et al., 2024). Therefore, rather than targeting only the SNP sites, we adopted a broad activation strategy aimed at encompassing the wider landscape of regulatory elements surrounding each SNP. To broadly activate these regions, we designed a multiplex gRNA library comprising four gRNAs per target CRE. Given the limitation associated with the uncertainty in identifying causal SNPs, we shifted our focus from SNP-specific transcriptional effects to the broader functional impact of activating the regulatory regions that harbor these SNPs. Through this approach, we aimed to identify downstream genes and altered biological pathways influenced by these regions, and to evaluate their potential relevance to the phenotype of interest. Accordingly, we sought to detect global transcriptional changes under sustained, rather than transient, activation. To achieve this, the gRNA constructs were stably integrated into the genome using the piggyBac transposon system (Ding et al., 2005). The piggyBac transposon system mediates random genomic integration of cargo sequences, including the gRNA expression cassette used in this study. Although integration sites cannot be precisely mapped, the use of bulk populations carrying randomly integrated constructs minimizes the influence of integration site–specific effects, as such variation is averaged across the entire population. In addition, the mock gRNA controls were delivered through the same random integration process, providing an appropriate reference for normalization. Given that our analyses were performed using bulk RNA-seq, localized effects of integration are unlikely to confound the observed transcriptional changes. This strategy has also been validated in previous CRISPRa and CRISPRi studies employing piggyBac-mediated gRNA delivery (Hazelbaker et al., 2020).

To investigate the downstream effects of activating these regions, we performed transcriptome profiling via bulk RNA-seq. We observed that activation of three distinct regulatory regions affected a shared set of biological pathways, including the MAPK signaling pathway, Cytoskeleton in muscle cells, Focal adhesion, and ECM-receptor interaction. The MAPK signaling pathway consists of a phosphorylation cascade mediated by serine/threonine kinases such as ERK, JNK, and p38, and it plays essential roles in muscle development, differentiation, and energy metabolism (Keren et al., 2006; Xie et al., 2018; Bengal et al., 2020). The Cytoskeleton in muscle cells, Focal adhesion, and ECM-receptor interaction pathways contribute to the organization of muscle tissue and interact to maintain structural stability and regulate muscle growth and differentiation (Henderson et al., 2025; Chen et al., 2023). Notably, these pathways are also key components of mechanotransduction, the cellular process that converts mechanical forces into biochemical signals. Mechanical stimuli transmitted through mechanotransduction activate intracellular signaling cascades, notably AMP-activated protein kinase (AMPK), which enhances glucose uptake and stimulates ATP synthesis to support the elevated energy requirements of muscle cells (Dawson et al., 2023; Kamal and Trombetta-Lima, 2025). ATP is a key molecule in the purine metabolic pathway, serving as a precursor of IMP and inosine (Uemoto et al., 2017). A previous study reported that focal adhesion and actin cytoskeleton organization are key pathways associated with IMP deposition, and identified thrombospondin-1 (*THBS1*), a gene related to these pathways, as being significantly correlated with IMP content in chicken muscle (Yu et al., 2023). Consistent with previous findings, our DEG analysis also detected increased expression of thrombospondin-related genes (*THBS1* and *THBS2*) in GW1 and GW2 (Supplementary Tables S4, 5). These results suggest that GW1 and GW2 non-coding SNP regions may influence the expression of specific genes, either directly or indirectly, thereby impacting downstream muscle-related pathways and potentially modulating purine metabolism indirectly through mechanotransduction-related signaling in chicken muscle.

We further analyzed gene expression changes near the SNP regions to assess their *cis*-regulatory activity. The activation of GW1 and GW3 led to increased expression of their direct neighboring genes, *DUSP8* and *FBXO3*, consistent with previous predictions that these regions could function as promoters. In contrast, activation of GW2 did not upregulate adjacent genes but induced the most significant transcriptional alterations. This intriguing result suggests that the GW2 enhancer region may modulate gene expression through alternative epigenetic mechanisms. Enhancers can regulate gene expression by forming chromatin loops or topologically-associated domains (TADs) that interact with promoters of proximal or distal genes on the same chromosome (Schoenfelder and Fraser, 2019). However, several studies have demonstrated that enhancers can engage in inter-chromosomal interactions through spatial proximity in the three-dimensional nuclear architecture (Maass et al., 2018; Moon et al., 2023; Tomikawa, 2024). Although our data did not directly capture such interactions, the broad transcriptional response associated with GW2 underscores the potential role of higher-order chromatin architecture in gene regulation. Incorporating Chromosome Conformation Capture (3C)-based high throughput techniques, such as Hi-C or Capture-C, in future studies could provide deeper insight into these regulatory mechanisms.

Transcript-level quantification analysis revealed that the GW1 region functions as the promoter for a short isoform of *DUSP8*, which encodes a truncated dual-specificity phosphatase 8. DUSP8 is a major negative regulator of phosphorylation-mediated signaling in the MAPK pathway and is predominantly expressed in the heart, brain, and skeletal muscle (Ding et al., 2019; Mutlak and Kehat, 2021). It suppresses the activation of multiple MAPK family members, including ERK1/2, JNK1/2, and p38, through dephosphorylation (Liu et al., 2016; Smeeton et al., 2016). In chickens, DUSP8-mediated inhibition of ERK1/2 phosphorylation has been shown to increase lipid accumulation and progesterone synthesis in granulosa cells (Sun et al., 2024). In humans, ERK phosphorylation increases IMP production by activating phosphoribosylformylglycinamidine synthase (PFAS), a key enzyme in the *de novo* purine synthesis pathway (Ali et al., 2020). These findings suggest that *DUSP8* may directly regulate the accumulation of purine nucleotides, such as IMP and inosine, by modulating ERK phosphorylation. This is consistent with our results, which suggest that regulation of *DUSP8* expression may influence the content of nucleotide-related compounds in skeletal muscle. Unlike the full-length protein, the short isoform of DUSP8 lacks the N-terminal rhodanese domain containing the kinase interaction motif (KIM), which is essential for substrate specificity. Such DUSP family proteins lacking the KIM motif are classified as atypical DUSPs (Lang and Raffi, 2019). Although the substrate specificity of atypical DUSPs remains poorly defined, they have been reported to interact with a wide range of targets, including MAPK family members, RNA, and scaffold proteins, suggesting broad physiological roles (Cho et al., 2017; Kincaid et al., 2018; Li et al., 2021). However, the functions of atypical DUSP8 remain unexplored in chickens. In addition, this short isoform transcript is expressed at higher levels than the long isoform in muscle tissue. These findings raise the possibility that non-canonical DUSP8 contributes to muscle physiology in chickens, emphasizing the importance of further functional characterization.

## 5 Conclusion

This study highlights the potential of CRISPRa as a powerful functional genomics tool for characterizing non-coding variants and its surrounding regulatory regions underlying complex traits in chickens. Our findings provide functional evidence that the non-coding GWAS variants we identified can act as regulatory elements influencing meat quality via muscle-related gene expression. RNA-seq analysis following CRISPRa-mediated activation of SNP-containing regions revealed downstream changes in biological pathways, including mechanotransduction in muscle and MAPK signaling, both of which are directly and indirectly linked to purine metabolism. Furthermore, we found that the most significant non-coding SNP region in GWAS functions as an alternative promoter for the expression of atypical *DUSP8*, which may influence the deposition of nucleotide-related compounds in chicken muscle. Overall, this study demonstrates the value of integrative functional genomics approaches for uncovering the regulatory roles of non-coding variants in avian species.

## Data Availability

The RNA-seq data are available in NCBI’s Sequence Read Archive (SRA) database with accession number PRJNA1273710 and PRJNA1253722.
