# Supplementary material for: From GWAS signal to function: targeted CRISPR activation enables functional characterization of non-coding SNPs in chickens
Source: Front Genome Ed. 2025 Oct 1;7:1662152. doi: 10.3389/fgeed.2025.1662152 (PMC12536144; doi:10.3389/fgeed.2025.1662152)
Supplement: Supplementary file 2 [file DataSheet1.docx]

Supplementary Material
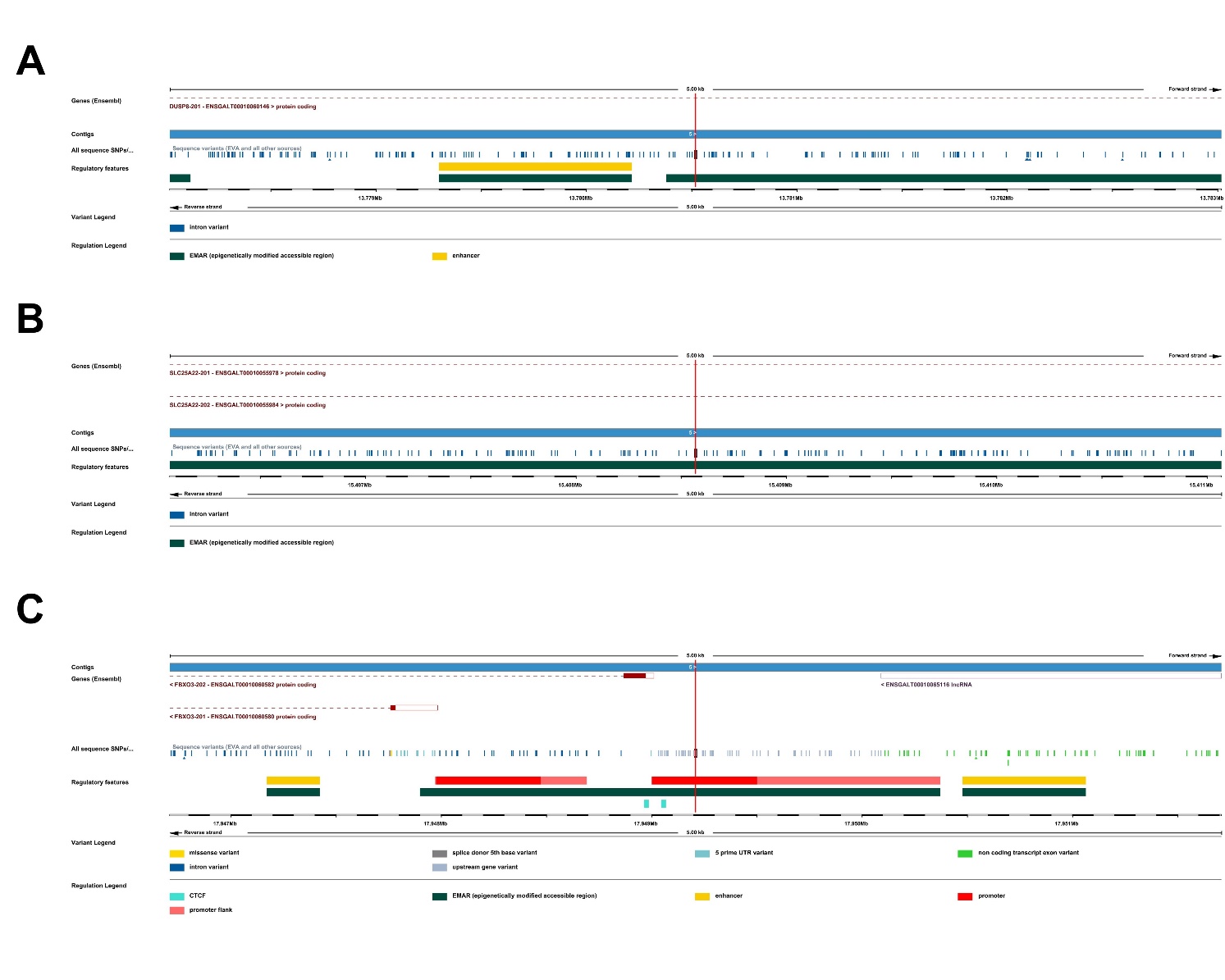


**Supplementary Figure S1**. Epigenomic features of non-coding SNP regions in Ensembl regulatory track (GRCg7b, release 113). (**A**) GW1 (rs316338889) (**B**) GW2 (rs313523098) (**C**) GW3 (rs317345807). Red vertical lines indicate the genomic positions of each non-coding SNP.


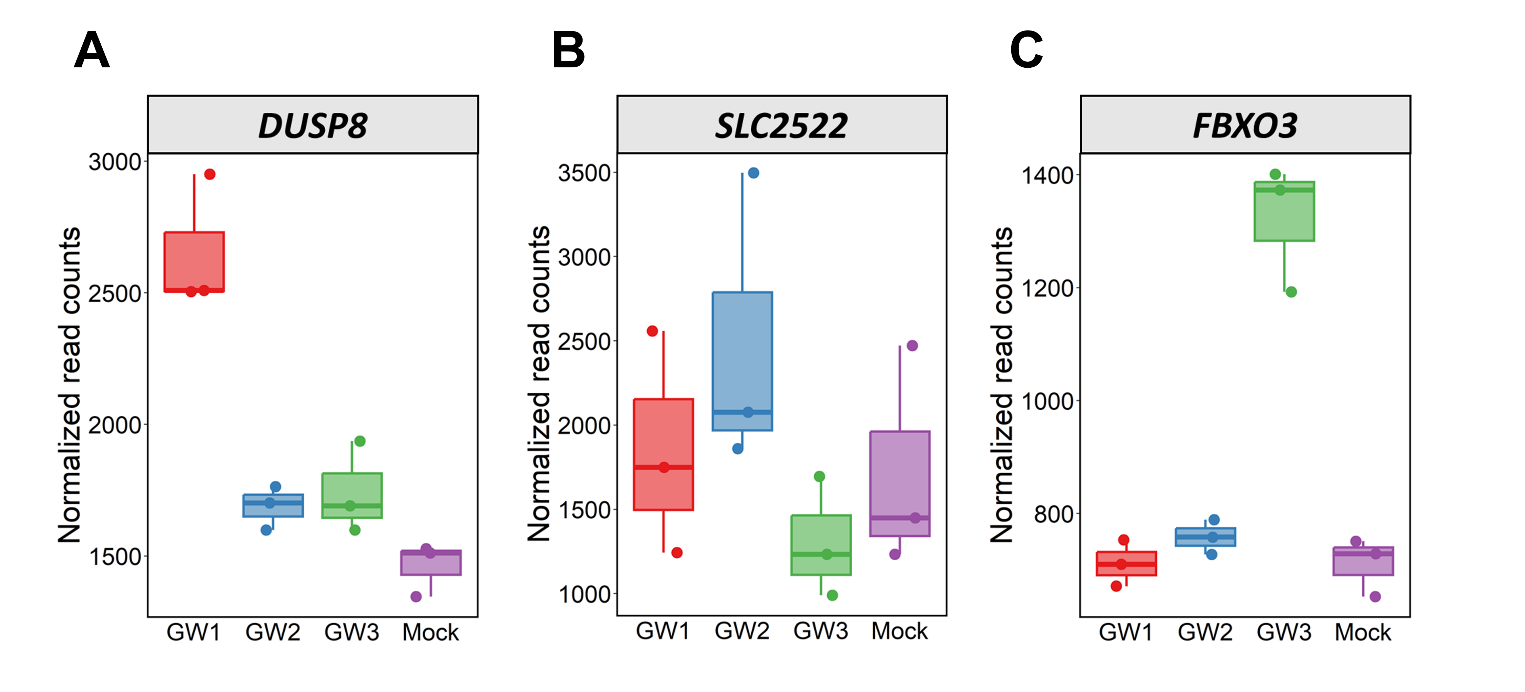


**Supplementary Figure S2**. Expression levels of SNP-harboring genes across GW1, GW2, and GW3 activation and mock conditions. (**A**) *DUSP8*. (**B**) *SLC25A22*. (**C**) *FBXO3*.
